# Proteomic Profiling of CSF Reveals Inflammatory Pathways Associated with Tau Neuropathology in Alzheimer's Disease

**DOI:** 10.1002/alz70856_106780

**Published:** 2026-01-08

**Authors:** Sofia Kinton, Simon Dujardin, Mikhail Levit, Ping‐Chieh Pao, Pablo Sardi, Bailin Zhang, James Dodge, Rajaraman Krishnan, Marianna Rizzo, Charlotte E. Teunissen, Henrik Zetterberg, Kaj Blennow, Gwendlyn Kollmorgen, Nancy Ramia, Sara B. Gomes Fernandes, Rejko Krüger, Olga Borejko, Dag Aarsland, Michele Hu, Thomas Klockgether, Kathrin Brockmann, Thomas Gasser, Frank Jessen, Annika Spottke, Wiesje M. van der Flier, Afina W. Lemstra, Betty M. Tijms, Giovanni B. Frisoni, Jakub Hort, Zuzana Nedelska, Claire Chevalier, Pieter Jelle Visser, Stephanie J. B. Vos

**Affiliations:** ^1^ Sanofi, Cambridge, MA, USA; ^2^ Sanofi, Boston, MA, USA; ^3^ Alzheimer Center Limburg, School for Mental Health and Neuroscience, Maastricht University, Maastricht, Netherlands; ^4^ Neurochemistry Laboratory and Biobank, Department of Clinical Chemistry, Amsterdam university medical center, Amsterdam, Netherlands; ^5^ Wisconsin Alzheimer's Disease Research Center, University of Wisconsin School of Medicine and Public Health, University of Wisconsin‐Madison, Madison, WI, USA; ^6^ Department of Psychiatry and Neurochemistry, Institute of Neuroscience and Physiology, the Sahlgrenska Academy at the University of Gothenburg, Mölndal, Sweden; ^7^ Department of Neurodegenerative Disease and UK Dementia Research Institute, UCL Institute of Neurology, Queen Square, London, United Kingdom; ^8^ Laboratory of Clinical Chemistry, Sahlgrenska University Hospital, Gothenburg, Sweden Institute of Neuroscience and Physiology, the Sahlgrenska Academy at the University of Gothenburg, Mölndal, Sweden; ^9^ Hong Kong Center for Neurodegenerative Diseases, Hong Kong, China; ^10^ Clinical Neurochemistry Laboratory, Sahlgrenska University Hospital, Mölndal, Sweden; ^11^ Department of Psychiatry and Neurochemistry, Institute of Neuroscience and Physiology, The Sahlgrenska Academy, University of Gothenburg, Mölndal, Sweden; ^12^ Roche Diagnostics GmbH, Penzberg, Germany; ^13^ Luxembourg Centre for Systems Biomedicine, University of Luxembourg, Esch‐sur‐Alzette, Luxembourg; ^14^ Transversal Translational Medicine, Luxembourg Institute of Health, Strassen, Luxembourg; ^15^ Parkinson Research Clinic, Centre Hospitalier de Luxembourg, Luxembourg, Luxembourg; ^16^ Institute of Psychiatry, Psychology and Neuroscience, King's College London, London, United Kingdom; ^17^ Centre for Age‐Related Medicine, Stavanger University Hospital, Stavanger, Stavanger, Norway; ^18^ Nuffield Department of Clinical Neurosciences, University of Oxford, Oxford, United Kingdom; ^19^ Oxford Parkinson's Disease Centre, University of Oxford, Oxford, United Kingdom; ^20^ German Center for Neurodegenerative Diseases (DZNE), Bonn, Germany; ^21^ Department of Neurology, University of Bonn, Bonn, Germany; ^22^ German Center for Neurodegenerative Diseases (DZNE), Tübingen, Germany; ^23^ Hertie Institute for Clinical Brain Research, Department of Neurodegenerative Diseases, University of Tübingen, Tübingen, Germany; ^24^ Alzheimer Center Amsterdam, Neurology, Vrije Universiteit Amsterdam, Amsterdam UMC location VUmc, Amsterdam, Netherlands; ^25^ Alzheimer Center Amsterdam, Neurology, Vrije Universiteit Amsterdam, Amsterdam UMC location VUmc, Amsterdam, Noord‐Holland, Netherlands; ^26^ Memory Clinic, Geneva University Hospitals, Geneva, Switzerland; ^27^ Memory Clinic, Department of Neurology, Motol University Hospital, Prague, Czech Republic; ^28^ Charles University, Second Faculty of Medicine and Motol University Hospital, Prague, Czech Republic; ^29^ Alzheimer Center Limburg, School for Mental Health and Neuroscience, Maastricht University, Maastricht, Limburg, Netherlands

## Abstract

**Background:**

Aggregation of amyloid beta (Aβ) and tau proteins is a hallmark of Alzheimer's disease (AD) and tauopathies. In AD, extracellular deposition of Aβ peptide precedes tau aggregation and the onset of neuroinflammatory processes. Both neuroinflammation and tau deposition are associated with synaptic loss, neurodegeneration, and cognitive decline. However, the precise sequence of events from amyloid deposition to tau aggregation, neuroinflammation, and neurodegeneration remains unclear. A cross‐sectional analysis of tau and other proteins in the cerebrospinal fluid (CSF) of AD patients can provide insight into the inflammatory and degenerative processes. Tau levels may be used as a proxy for disease progression, and we hypothesize that the expression of neuroinflammatory markers will be modulated in response to increasing tau aggregation.

**Method:**

To investigate this, we collaborated with the EPND‐biomarker consortium to analyze over 150 human CSF samples from AD and control patients. We measured levels of Aβ1‐40, Aβ1‐42, pTau181, and total tau, and conducted proteomic profiling using the O‐link and SomaScan platforms. Next, AD patients were stratified into Aβ+Tau+ and Aβ+Tau‐ groups to identify biomarkers associated with Aβ and/or tau aggregation.

**Result:**

As expected, inflammation markers were elevated in AD CSF samples compared to controls. Interestingly, we observed distinct correlations between these markers and amyloid versus tau pathology biomarkers. Approximately 10% of the measured CSF proteins (∼250 proteins) showed significant differences (*p* < 0.05) between AD and control samples. CSF samples with elevated tau levels exhibited altered levels of proteins involved in the complement cascade, cytokine‐cytokine receptor interactions, cell adhesion, and vesicular transport.

**Conclusion:**

Increased inflammation markers were observed in AD. Proteins linked to innate immune response and synaptic loss were significantly altered in relation to tau aggregation. These findings are crucial for guiding therapeutic strategies for neurodegenerative diseases and assessing their efficacy.

